# Medical and surgical treatment of haemorrhoids and anal fissure in Crohn’s disease: a critical appraisal

**DOI:** 10.1186/1471-230X-13-47

**Published:** 2013-03-11

**Authors:** Stefano D'Ugo, Luana Franceschilli, Federica Cadeddu, Laura Leccesi, Giovanna Del Vecchio Blanco, Emma Calabrese, Giovanni Milito, Nicola Di Lorenzo, Achille L Gaspari, Pierpaolo Sileri

**Affiliations:** 1 Department of Surgery, University Hospital Tor Vergata, Viale Oxford 81, Rome 00133, Italy; 2 Department of Gastroenterology, University Hospital Tor Vergata, Rome, Italy; 3 Department of Internal Medicine, Catholic University, Rome, Italy

**Keywords:** Haemorrhoids, Anal fissure, Crohn’s disease, Botox, Surgery

## Abstract

**Background:**

The principle to avoid surgery for haemorrhoids and/or anal fissure in Crohn’s disease (CD) patients is still currently valid despite advances in medical and surgical treatments. In this study we report our prospectively recorded data on medical and surgical treatment of haemorrhoids and anal fissures in CD patients over a period of 8 years.

**Methods:**

Clinical data of patients affected by perianal disease were routinely and prospectively inserted in a database between October 2003 and October 2011 at the Department of Surgery, Tor Vergata University Hospital, Rome. We reviewed and divided in two groups records on CD patients treated either medically or surgically according to the diagnosis of haemorrhoids or anal fissures. Moreover, we compared in each group the outcome in patients with prior diagnosis of CD and in patients diagnosed with CD only after perianal main treatment.

**Results:**

Eighty-six CD patients were included in the study; 45 were treated for haemorrhoids and 41 presented with anal fissure. Conservative approach was initially adopted for all patients; in case of medical treatment failure, the presence of stable intestinal disease made them eligible for surgery. Fifteen patients underwent haemorrhoidectomy (open 11; closed 3; stapled 1), and two rubber band ligation. Fourteen patients required surgery for anal fissure (Botox ± fissurectomy 8; LIS 6). In both groups we observed high complication rate, 41.2% for haemorrhoids and 57.1% for anal fissure. Patients who underwent haemorrhoidectomy without certain diagnosis of CD had significantly higher risk of complications.

**Conclusions:**

Conservative treatment of proctologic diseases in CD patients has been advocated given the high risk of complications and the evidence that spontaneous healing may also occur. From these preliminary results a role of surgery is conceivable in high selected patients, but definitve conclusions can’t be made. Further randomized trials are needed to establish the efficacy of the surgical approach, giving therapeutic recommendations and guidelines.

## Background

The original description of Crohn’s disease (CD) in 1932 included only “regional ileitis” and not perianal lesions. However from 1938, when Penner and Crohn first reported a perianal fistula in an affected patient, it became clear that the perianal pathology represented a common medical problem in CD patients [1,2].

The actual incidence is not known, being reported in literature as low as 3.8% and as high as 61–80% [3-6]. In 64–68% of patients perianal disease occurs either concurrently or after the diagnosis of intestinal disease [7,8]; however in 20–36% the perianal disease precedes the intestinal disease [3,7].

The prevalence increases as the disease progresses distally, particularly if the rectum is affected; moreover, in absence of rectal inflammation, anal manifestations have a better outcome [3,9].

The presence of perianal CD is associated with a more disabling natural history, with increased extraintestinal manifestations and greater steroid resistance [10,11]. Furthermore these subjects were found at greater risk of proctectomy, reported to be as high as 5% at first presentation of perianal disease, increasing to 8% after 10 years and doubling after 20 years from diagnosis [12-14].

The majority of Authors focused the attention on natural history, treatment and complications of anal sepsis and fistulae, because of their higher incidence and surgical implications. On the contrary, extensive analysis about the management of haemorrhoids and anal fissure has been overlooked, although they can represent an important problem in CD treatment [4,5,8].

At present, there is still no consensus in the scientific community on the exact indications of surgery in CD patients presenting with anal fissure or haemorrhoids, mainly due to scant data in literature.

In this report we made a retrospective analysis of our longitudinal prospective data on symptoms, medical therapy results, surgical treatment and complications in a dedicated colorectal unit in a university hospital.

## Methods

Clinical records of patients affected by perianal disease were routinely and prospectively entered in a database between October 2003 and October 2011 at the Department of Surgery, Tor Vergata University Hospital in Rome. We retrospectively reviewed data on CD patients included in the study, treated either medically or surgically for anal fissures or haemorrhoids.

Exclusion criteria were the presence of concomitant suppurative disease, perianal fistula or cancer. During the first visit everyone underwent a baseline evaluation which included anamnesis, Wexner continence score, clinical examination of the perineum and rectum by means of digital exploration and anoscopy. Patients with evidence or risk of incontinence scheduled for surgery underwent anal manometry.

Medical and/or surgical treatments were undertaken in agreement with gastroenterologists who followed patients for the specific pathology, in order to evaluate the best time for surgery.

Institutional Review Board (IRB) approval, in compliance with the Helsinki Declaration, has been obtained from the Ethic Committee of our Institution (“Comitato Etico Indipendente Fondazione Policlinico Tor Vergata”).

The prospectively recorded data included demographics, clinical presentation of perianal pathology, type and results of medical and/or surgical treatment, postoperative course, complications, recurrence and symptoms at follow-up.

Analysis of the results was made dividing the patients in two groups, accordingly with the diagnosis of haemorrhoids or anal fissure. Moreover, we compared in each group the outcomes between patients with diagnosis of CD made prior or after perianal main treatment.

The diagnosis of CD was established in agreement with the European Crohn’s and Colitis Organisation (ECCO). Patients underwent a full evaluation by means of clinical, laboratory, endoscopic, radiologic and histologic investigations. The grading of the pathology was established mainly using the Crohn’s Disease Activity Index (CDAI), but also evaluating other parameters like the level of the C-reactive protein (CRP) and the length of bowel involvement.

Statistical analysis was performed with the software Statgraphics Plus (Manugistics, Rockville, Maryland); Student’s *t* test was used for continuous variables and Fisher exact test for categorical variables.

Results are expressed as median values and range if not stated otherwise. All tests were double-sided and the level of statistical significance was set at a *p* value of less than 0.05.

## Results

Between October 2003 and October 2011, we identified eighty-six patients with diagnosis of CD suffering from anal fissure or haemorrhoids, treated either medically and/or surgically at our Department of Surgery, who fulfilled the study criteria. Median follow up was 37 months (range 3–99).

Overall, 45 CD patients were evaluated and followed up with diagnosis of haemorrhoids and the remaining 41 CD patients for anal fissure. Table 1 shows demographics and presenting symptoms in the two groups.

**Table 1:** Demographics and presenting symptoms

	**Haemorrhoids (n = 45)**	**Anal fissure (n = 41)**
**Age***(yrs)*	39 (21–60)	41 (18–64)
*median (range)*		
**Male/Female ratio**	1.8:1	2.1:1
**CD diagnosis** (%)	24 (53.3%)	22 (53.7%)
-ileum	21	18
-colon	2	2
-both	1	2
**Symptoms** (%)		
-bleeding	91%	88%
-prolapse	62%	-
-anal discomfort	54%	60%
-thrombosis	19%	-
-pain	-	81%
-sentinel pile	-	58%

In Table 2 is presented a patients resume’ of medical and surgical treatments, showing the categories of subjects who had or didn’t have definitive diagnosis of CD at the time of the main treatment.

**Table 2:** Medical and surgical treatments resumè (n = 86)

	**Conservative treatment (n = 55)**	**Surgical treatment (n = 31)**
**Without CD diagnosis (n = 40)**		
Haemorrhoids	13	8
Anal Fissure	11	8
**With CD diagnosis (n = 46)**		
Haemorrhoids	15	9 (2 rubber band ligation)
Anal Fissure	16	6

### Haemorrhoids

Conservative approach was initially adopted in all the patients; in absence of diarrhoea, it included high fibre diet, fibre supplements and oral fluids intake in order to produce soft, well-formed stools and regular bowel movements. Warm Sitz baths were also suggested. Oral diosmin was added to this first line therapy if symptoms persisted after 12 weeks or in case of thrombosis at the first outpatient visit. Moreover it represented the main treatment in CD patients with frequent diarrhoea (n = 5).

This conservative approach failed in 17 (37.8%) out 45 patients. Indication to surgical treatment in the nine patients with known CD at the time of evaluation was given only in case of stable intestinal disease, without need of steroid medications and with CDAI < 150.

Table 3 resumes types of surgical approach and overall complications in this group of patients; moreover the statistical analysis between the two subgroups is reported.

**Table 3:** Treatments and postoperative complications after failure of conservative management of haemorrhoids (n = 17)

	**Without CD diagnosis (n = 8)**	**With CD diagnosis (n = 9)**	***p***
Open haemorrhoidectomy	5	6	n.s.
Closed haemorrhoidectomy	2	1	n.s.
Stapled haemorrhoidopexy	1	0	n.s.
Rubber Band Ligation	0	2	n.s.
**Postoperative Complications**	6	1	0.015
	Bleeding (2)	Bleeding	
	Fissure (2)		
	Sepsis (2)		

Mean time to complete healing after surgery was 38 ± 8 days.

The most common complication was postoperative bleeding, observed in 3 (17.6%) out 17 patients, during the first four days postoperatively. One bleeding was self-limiting, while the other two required Emergency Room visits, during which local compression with hemostatic gauze (Tabotamp, Johnson & Johnson) was effective.

During the follow up we observed two (11.8%) postoperative anal fissures, effectively treated with topic glycerin trinitrate (GTN) 0.4% for eight weeks. In two cases (11.8%) perianal sepsis was detected one month and forty days after surgery, in the form of abscess and intersphinteric fistula close to one site of haemorrhoid excision. These patients were then successfully treated by drainage and fistulotomy.

### Anal fissure

Based on the anatomic position in the anal canal, fissures were posterior in 23 (56.1%) patients, anterior in 9 (22.0%), lateral in 6 (14.6%) and both (anterior and posterior) in 3 (7.3%).

The first line therapy was medical, with topic application of calcium channels blockers or GTN 0.4% ointment twice a day for 8 weeks. The choice of one of these two options was made mainly according to coexisting medical conditions or evaluating the potential side effects (i.e. heart disease, hypertension, headache).

Conservative treatment was effective in 27 patients (65.8%). Indication for the operative procedure in the six patients with known CD was given only if the disease was in a remission state (CDAI < 150), with no steroid therapy.

Table 4 summarizes the surgical approaches and complications in these patients; furthermore the statistic results are shown.

**Table 4:** Treatments and postoperative complications after failure of conservative management of anal fissure (n = 14)

	**Without CD diagnosis (n = 8)**	**With CD diagnosis (n = 6)**	***p***
Botox/Botox + Fissurectomy	0/2	4/2	n.s.
Lateral Internal Sphincterotomy	6	0	0.009
**Postoperative Complications**	5	3	n.s.
	Non Healing (3)	Recurrence (2)	
	Recurrence (1)	Non Healing (1)	
	Fistula (1)		

All the six patients with CD diagnosis underwent botox injection in the internal anal sphincter (IAS), and in two cases fissurectomy. In the other subgroup, lateral internal sphincterotomy (LIS) was the most common surgery (75%), in addition to botox and fissurectomy in two cases (p = 0.009).

Mean time to complete healing was 18 ± 5 days after Botox + Fissurectomy and 25 ± 7 days after LIS.

Overall complication rate was 57.1% (n = 8), with similar incidence in the two subgroups (p > 0.05). The most common was the occurrence of a non-healing wound (n = 4; 28.6%), two after LIS and two after Botox + Fissurectomy. These patients were than treated with conservative therapy and eventually reached the cure after several weeks from surgery. Moreover we observed three recurrences, treated by GTN 0.4% ointment. One patient suffered from a trans-sphincteric anal fistula after LIS, which healed without incontinence after placement of a cutting seton.

## Discussion

In patients without a diagnosis of IBD, after failure of medical approaches, aggressive treatment of haemorrhoids and anal fissure is usually uneventful. On the other hand, the management of these pathologies in subjects with CD is though to be hazardous, despite literature data are surprisingly scant. This is due to the report of significant complications, including sepsis, stenosis, fistulas, faecal incontinence and non-healing wounds even after simple procedures such as haemorrhoidectomy or fissurectomy.

In this paper we report the results of a retrospective analysis of longitudinal prospective data of patients with CD treated with conservative and surgical approaches for haemorrhoids and anal fissure, comparing the outcomes between those patients whose CD was discovered before and those in whom CD diagnosis was made after perianal main treatment.

We are aware of some limitations of the present study, primarily because it is a case series, and our aim is not to give therapeutic recommendations. However these data may represent a starting point for further studies in the form of randomized trials, and therefore we believe that some considerations can be made.

### Haemorrhoids

Haemorrhoids are relatively uncommon in CD patients, who usually report few symptoms. The estimated incidence is about 7%, which seems to be significantly lower than the general unaffected population (24%) [15]. However this anal problem could be underestimated, because of a bias due to the higher attention paid to the other clinical features of CD.

Historically surgery was firmly obstructed; in one of the first articles on this subject, Jeffrey et al. concluded that absolutely no surgical treatment should be given to CD patients, reporting severe complications in more than half of them [16].

On the contrary, Wolkomir and Luchtefeld successively published their series in which 88% of CD patients, who underwent surgery for symptomatic haemorrhoids, healed without any complication. They showed that, when the intestinal disease is quiescent and after failure of conservative treatments, a surgical option may be offered in selected cases [12].

In our experience, conservative treatment was effective in more than 60% of patients. Operative approach was required because of persisting symptoms, mainly bleeding (91%) and prolapse (62%). Indication to surgery was not influenced by the diagnosis of IBD, but only by the clinical condition; in fact, the two subgroups underwent surgical treatment homogeneously.

Usually we preferred the “open haemorrhoidectomy”, in both subgroups; however in patients with IBD diagnosis, we also performed the rubber band ligation, associated with less operative risks, but effective in both of our cases. Actually we decided to include the latter treatment in the operative group, even if sometime could be considered a kind of conservative procedure. This was done to evaluate the efficacy of the medical therapy without any form of invasive technique, considering the potential effects on the intestinal mucosa and the anal canal.

Furthermore needs to be remarked that between various Countries there are different treatment protocols for haemorrhoids. There is indeed a discrepancy in the use of phlebotonics, usually not prescribed in Northern Europe and United States in favour of other medical therapies; also the spread of the rubber band ligation is much greater in other centres compared to our group, often as treatment of choice for this pathology.

If the surgeon knew that IBD was present, haemorrhoidopexy was never indicated, since literature data have shown increased risk of life-threatening complications, mainly sepsis and bleeding, after SH compared to conventional haemorrhoidectomy [17]. Nevertheless, the only patient of the non-IBD subgroup at the time of surgery did not incur in any postoperative problem.

After surgery seven patients (41.2%) experienced some complications; if we excluded the rubber band ligation, in which there isn’t tissue excision and not associated with complications in our series, the rate raised to 46.7%. This incidence is significantly higher compared to excisional haemorrhoidectomy or stapled haemorrhoidopexy in the unaffected population, reported in literature to be between 15% and 25% [18,19]. Likewise, from the lately published experience of our group, the incidence of complications after surgery was about 20%, in agreement with literature data [20].

Moreover in the subgroup with no IBD diagnosis at that time, complications were significantly more frequent than in the other one. This is probably due to the fact that we performed a more conservative and careful procedure if CD was known, and confirms the knowledge of the high risk of surgery in this category.

In our series the most common complication was postoperative bleeding; particularly, patients have to be informed about the possibility of a haemorrhage. This usually manifests during the first hours or few days after surgery, and may need a hospital treatment.

To date, proctectomy was not required in any patient and we believe that it is not an inevitable outcome after haemorrhoid surgery in these subjects.

Based on these results it seems that surgery, in the form of excisional haemorrhoidectomy or rubber band ligation, may have a role after failure of medical treatments. However more data are needed to confirm these outcomes and to correlate them with complications and disease activity.

### Anal fissure

Anal fissures are more common than haemorrhoids in CD patients and often associated with other perianal pathologies. Wolff et al., over a follow-up period of 26 years, reported a 35% incidence of anal fissures [13]; Lockhart-Mummery at St. Mark’s hospital reported a rate of 59% [21].

Although their prevalence in the general population is not easily assessable, anal fissures seem to be more frequent than in unaffected subjects (as many as one out of five people develops an acute or chronic fissure during lifetime) [22,23].

Unlike typical fissures, these seem to be secondary to the direct ulceration caused by the disease process rather than the increased internal sphincter pressure; they can be locally aggressive, progressing to form deep ulcers with granulating bases and overhanging skin edges.

Differently from the general population, aberrant positions are common; multiple and lateral fissures were reported in 32-33% and 9-20% of patients respectively [24,25]. This is confirmed in our data in which, although more than half of the patients had a posterior fissure, 43.9% of them presented with unusual location.

Whereas in non-IBD subjects anal fissures are usually symptomatic, in CD patients pain, bleeding and anal discomfort have been reported in only 44-70% of cases, and they may be hence completely quiescent. In our series the majority of subjects reported one of the typical symptoms, but asymptomatic patients with diagnosis of anal fissure were encountered.

Although conservative medical therapy or simple observation is also indicated for the management of anal fissures, it should be considered that unhealed fissure may progress to fistula or abscess in up to 20% of the cases.

Common local anorectal procedures such as sphincterotomy or anal dilation are infrequently performed in CD patients, due to the perception of putting the patients at risk of incontinence, as they frequently have an underlying diarrhea state and are at significant risk of requiring additional anal surgery in the future. Despite good results after anal dilation and stretch have been reported in erratic series, we agree with Fleshner et al. that dilation of the sphincter should be avoided in CD, not only because of suboptimal healing of the fissure but also to avoid uncontrolled trauma to diseased anal mucosa with potential development of secondary infection or fistula [24-26].

Regarding the use of lateral internal sphincterotomy, it has been advocated to treat selected CD patients, but literature data is limited and based on small series. Wolff et al. suggested that painful fissures should be converted to a painless state by sphincterotomy [13]. Accordingly, Cohen et al. stated that a limited sphincterotomy may be performed after failure of all medical approaches [27].

Wolkomir and Luchtefeld reported anal fissure healing in about 90% of CD patients after surgery [12]. These results were also confirmed by Fleshner et al. with longer-term healing after surgical treatment in CD patients, who highlighted also the 25% risk of developing an abscess or a fistula from the base of the fissure, if they did not undergo LIS after failure of conservative treatment [25].

In our series, conservative medical therapy failed in about 34% of patients, without statistic difference between the two subgroups with or without diagnosis of IBD at that time. As for haemorrhoids, the presence of CD modified the surgical approach. We preferred to address those patients to botox injection in the internal anal sphincter, to best avoid the risk of incontinence following LIS. At the mean follow-up we did not have cases of incontinence, probably because always during this surgery we performed the least sphincter division to relax the apparatus. However more than half of the patients developed some complications after surgery, mainly difficulties in wound healing, confirming the higher risk compared to the general population. Indeed in literature complication rates range from 7% to 42%, and in our experience in unaffected subjects, it is about 10% [28,29].

According to these results and literature data, although not randomized, it could be argued a judicious choice of the surgical option in patients non-responding to conservative therapies. The idea would be to create small wounds, minimizing the damage to the mucosa and the external sphincter, and a closed subcutaneous LIS seems to be appropriate. Fissurectomy might be considered only when the edges of the fissure are densely fibrotic and are unlikely to heal after sphincterotomy alone. Botox injection could be an alternative to LIS, avoiding damages to the sphincter apparatus, risk of incontinence, and reducing wound healing complications.

## Conclusions

The occurrence of haemorrhoids and anal fissure associated to Crohn’s disease has to be considered. The main treatment of these conditions should be a medical therapy. From the preliminary results of this study it is conceivable, in case of failure, a role of surgery in high selected patients, always evaluating the risk of postoperative complications; however definitive conclusions can’t be made. Further studies, primarily randomized trials, are needed in order to establish the efficacy of the surgical approach, giving therapeutic recommendations and guidelines.

## Abbreviations

CD: Crohn’s Disease; IBD: Inflammatory Bowel Disease; SH: Stapled Haemorrhoidopexy; GTN: Glycerin Trinitrate; IAS: Internal Anal Sphincter; LIS: Lateral Internal Sphincterotomy.

## Competing interests

The authors declare that they have no competing interests.

## Authors’ contributions

SD, PS, LL: study design, analysis and interpretation of data, manuscript drafting. LF, FC, GDV, EC: collection of data, literature review, analysis of data. GM, NDL, ALG: important intellectual content, critical review and final approval. All authors read and approved the final manuscript.

## Pre-publication history

The pre-publication history for this paper can be accessed here:

http://www.biomedcentral.com/1471-230X/13/47/prepub
